# Decreased Thyroxine Levels during rhGH Therapy in Children with Growth Hormone Deficiency

**DOI:** 10.3390/jcm10215100

**Published:** 2021-10-30

**Authors:** Ewelina Witkowska-Sędek, Anna Małgorzata Kucharska, Małgorzata Rumińska, Monika Paluchowska, Beata Pyrżak

**Affiliations:** Department of Paediatrics and Endocrinology, Medical University of Warsaw, 02-097 Warsaw, Poland; ewelina.witkowska-sedek@wum.edu.pl (E.W.-S.); malgorzata.ruminska@wum.edu.pl (M.R.); monika.paluchowska@wum.edu.pl (M.P.); beata.pyrzak@wum.edu.pl (B.P.)

**Keywords:** thyroid function alterations, growth hormone deficiency (GHD), recombinant human growth hormone (rhGH) therapy, growth response, children

## Abstract

Background: Hypothyroidism in children leads to growth retardation. However, there is some evidence that recombinant human growth hormone (rhGH) therapy could suppress thyroid function. The most common observation in rhGH-treated patients is a decrease in thyroxine levels, which is reported as transient, but the studies in the field are inconsistent. We aimed to evaluate thyroid function in initially euthyroid children with idiopathic isolated GH deficiency during long-term rhGH therapy and to determine who is at a higher risk of thyroid function alterations during the therapy. Methods: The study group consisted of 101 children treated with rhGH for at least three years. Serum TSH and fT4 levels were determined at baseline, after the first six months and after each full year of therapy. The associations between changes in thyroid hormone levels during rhGH therapy and GH deficit, insulin-like growth factor-1 levels and growth response were investigated. Results: A significant decrease in fT4 levels (*p* = 0.01) was found as early as after the first six months of rhGH therapy. This effect persisted in the subsequent years of treatment without any significant changes in TSH values and tended to be rhGH dose related. Children with a greater fT4 decrease after the initiation of rhGH therapy were older, had higher bone age and responded to that therapy worse than children with lower fT4 changes. Conclusions: Our study revealed a long-term decrease in fT4 levels during rhGH therapy in initially euthyroid GHD children. The decrease in fT4 levels was associated with a lower growth response to rhGH therapy.

## 1. Introduction

One of the main symptoms of hypothyroidism in children, irrespective of its etiology, is growth impairment. However, there is some evidence that recombinant human growth hormone (rhGH) therapy could suppress thyroid function [1,2,3,4,5,6,7,8,9,10,11,12,13,14,15]. The increase in peripheral thyroxine (T4) to triiodothyronine (T3) conversion seems to be the main mechanism of those alterations [16,17,18,19,20,21], but the unmasking of central hypothyroidism after the initiation of rhGH has also been reported [22,23,24,25,26]. Unfortunately, the analyses in the field give inconsistent findings, resulting, in our opinion, from the heterogeneity of the study groups, the scarce number of enrolled patients, diversity in the indications for rhGH therapy and, usually, too short follow-ups. The most common finding in patients treated with rhGH is a significant decrease in free thyroxine (fT4) levels, which is considered as transient and observed mainly in the first year of therapy [16,18,21,27]. In GH-deficient children, a persistent decrease in fT4 levels due to rhGH therapy could result in a significant reduction in height velocity (HV) and, consequently, in deterioration of the growth response to the treatment. In those cases, levothyroxine (L-thyroxine) supplementation should be taken into account as a possible way to achieve the optimal growth effect of rhGH therapy [27]. The clinical significance of thyroid hormone changes during rhGH therapy seemed to be underappreciated despite many authors agreeing that the regular monitoring of thyroid function during rhGH therapy is needed [5,13,14,22,23,24,27,28,29]. 

The aim of our study was to evaluate the alterations in thyroid-stimulating hormone (TSH) and fT4 serum levels in initially euthyroid children with idiopathic isolated growth hormone deficiency (GHD) during long-term rhGH therapy. 

## 2. Materials and Methods

The present study included data of 101 children aged from 2.92 to 15.08 years treated with rhGH for isolated GHD. In most cases (87 out of 101 children), the observation period involved the first four years of rhGH therapy, whereas in 14 children, it lasted three years. GHD was defined as a peak GH level below 10 ng/mL in two stimulatory tests (clonidine/arginine/glucagon) and one test of spontaneous GH secretion during physiological sleep at night. Organic lesions of the hypothalamic–pituitary region were excluded in magnetic resonance imaging in all individuals before the initiation of rhGH therapy. All children were initially euthyroid and had a negative history of thyroid function abnormalities, and none of them had been treated with L-thyroxine either in the past or at the initiation of rhGH therapy. All the data were collected from medical records retrospectively (Department of Paediatrics and Endocrinology, Medical University of Warsaw, Poland), but all measurements of biochemical and anthropometric parameters evaluated in the analysis were in accordance with the Polish recommendations for rhGH therapy monitoring. We evaluated anthropometric and biochemical parameters at baseline and after the first six months and each full year of rhGH therapy. Anthropometric measurements were expressed as standard deviation scores (SDS) for chronological age (height) or for height age (weight and body mass index—BMI). Blood samples were taken at fasting. We analyzed serum TSH and fT4 levels (both measured by immunofluorescence assays using an ARCHITECT i1000SR, Abbot) and GH and insulin-like growth factor-1 (IGF-1) levels (both measured by immunoassay using an IMMULITE 2000 Xpi analyzer, Siemens, Munich, Germany ). IGF-1 values were normalized for sex and bone age and were presented as SDS according to the data supplied by the manufacturer (Siemens Healthcare Diagnostics Inc.). The peak GH level was defined as the highest GH concentration in diagnostic tests. Bone age was evaluated according to the Greulich and Pyle method [30] at baseline and after each full year of treatment. 

Statistical analysis was performed using c 13.3 (TIBCO Software Inc., Palo Alto, CA, USA). The normality of data distribution was checked by the Shapiro–Wilk test. Data were reported as means ± standard deviation (SD) or median with interquartile ranges. The baseline and treatment values of the same parameter were compared using the repeated measures ANOVA with Bonferroni post hoc test (parametric data) or using the Friedman test with post hoc comparisons (non-parametric data). Correlations between variables were evaluated using the Pearson correlation coefficient (parametric data) or Spearman correlation analysis (non-parametric data). A *p*-value < 0.05 was considered significant. 

## 3. Results

The characteristics of the baseline and treatment anthropometric parameters, biochemical parameters, bone age and rhGH doses administered in each year of therapy are presented in Table 1. The characteristics of changes in height SDS, IGF-1 SDS and fT4 values after the initiation of rhGH therapy are presented in Table 2. After the initiation of rhGH therapy, simultaneously with a significant reduction in height deficit (height SDS) resulting from an increase in HV coinciding with an increase in IGF-1 SDS values, we found that fT4 levels significantly decreased compared to baseline. The reduction in fT4 levels was observed as early as after the first six months of rhGH therapy (*p* = 0.01), and then fT4 levels plateaued for the next two years (*p* = 0.01 vs. baseline both after the first and the second year of rhGH therapy). After the third and fourth full year of treatment, we noticed a further decrease in fT4 levels compared to the values at baseline (*p* = 0.01) and after six months and the first year of therapy (*p* = 0.01 for both). Serum fT4 levels below the lower limit of the range were found in a small percentage of the studied children—in two individuals after the first six months of therapy, in three children after the first year, in another three after the second, in yet another three after the third and in two children after the fourth year of therapy. Only one patient received L-thyroxine substitution. In the remainder of the above-mentioned group, the fT4 level was slightly reduced in one measurement in each patient, not lower than 0.74 ng/dL, and then at the subsequent checkpoints, it was found to be within the lower limit of the range. Although changes in fT4 levels (ΔfT4) were not accompanied by significant changes in TSH levels vs. baseline values, we observed that TSH values fluctuated and reached the maximum level after the first year of rhGH therapy. We found that TSH levels measured after the first year of treatment were significantly higher than the values after the second (*p* = 0.01), the third (*p* = 0.01) and the fourth (*p* = 0.01) full year of rhGH therapy. 

**Figure 1:**
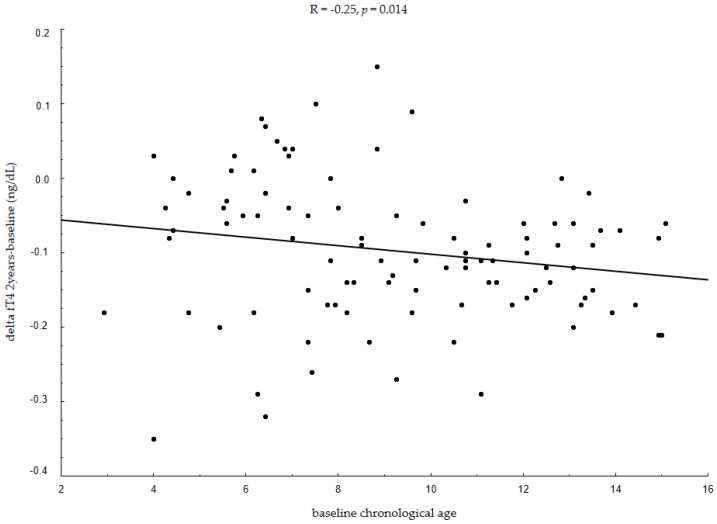
Associations between changes in fT4 levels in the first two years of rhGH therapy and baseline chronological age. fT4—free thyroxine; rhGH—recombinant human growth hormone; R—Spearman’s correlation coefficient.

In a further analysis, we attempted to determine which baseline parameters influenced the decreases in fT4 levels observed after the initiation of rhGH therapy. We found that changes in fT4 levels, both in the first (ΔfT4 1year-baseline) and the second year (ΔfT4 2years-baseline) of treatment, were negatively associated with baseline chronological age (R = −0.21, *p* = 0.035; R = −0.25, *p* = 0.014 (Figure 1), respectively) and baseline bone age (R = −0.20, *p* = 0.047; R = −0.22, *p* = 0.026, respectively). Older children and children with a higher bone age had a greater decrease in fT4 levels during rhGH therapy. We did not find any correlations between ΔfT4 after the initiation of rhGH therapy and baseline height deficit, baseline HV, baseline nutritional status (weight SDS or BMI SDS) or with baseline GH deficit defined as the maximum GH peak in diagnostic tests and as baseline IGF-1 SDS values.

**Figure 2:**
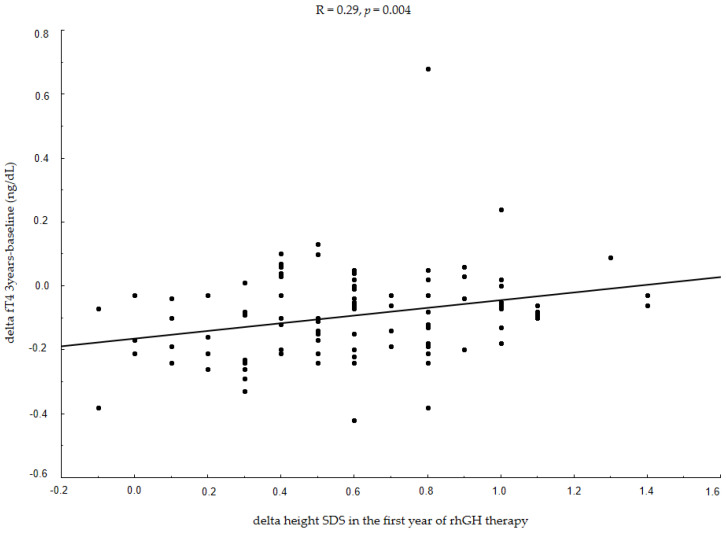
Associations between changes in fT4 levels in the first three years of rhGH therapy and growth response in the first year of therapy. fT4—free thyroxine; rhGH—recombinant human growth hormone; SDS—standard deviation score; R—Spearman’s correlation coefficient.

The next step in our analysis was to search for correlations between ΔfT4 during rhGH therapy and the response to that therapy defined as a reduction in height deficit (Δheight SDS) and an increase in IGF-1 SDS (ΔIGF-1 SDS). We found that children with a good growth response to rhGH therapy (higher Δheight SDS) had lower fT4 reduction during treatment. The changes in fT4 levels in the first three years of therapy (ΔfT4 3years-baseline) were significantly associated with a reduction in height deficit vs. baseline values (Δheight SDS) in the first (R = 0.29, *p* = 0.004, Figure 2), the second (R = 0.24, *p* = 0.018) and the third (R = 0.20, *p* = 0.043) year of therapy. 

We established that changes in fT4 levels within the first three and four years of rhGH therapy were related to changes in IGF-1 SDS (Table 3).

We also investigated whether there were any associations between changes in fT4 levels after the initiation of rhGH therapy and increases in bone age in the same period and between changes in fT4 levels and rhGH doses, which were administered in each year of treatment, but we did not find any significant relationships. We did observe almost statistically significant negative correlations between ΔfT4 in the first year of therapy and rhGH doses in that period (*p* = 0.06) and ΔfT4 2years-baseline and rhGH doses administered in the second year of treatment (*p* = 0.09), which could suggest that children with higher fT4 decreases during treatment were treated with higher rhGH doses. 

## 4. Discussion

Our results indicate that rhGH therapy in GH-deficient initially euthyroid children affects thyroid function, especially fT4 levels. Free T4 levels decreased significantly after the initiation of rhGH therapy, while TSH values fluctuated but did not change significantly compared to baseline levels. Changes in fT4 levels were long term and persisted in the subsequent years of rhGH therapy, with fT4 levels usually reaching the lower limit of the range or falling slightly below the normal range. Moreover, the variation in fT4 values seemed to be rhGH dose dependent and was more pronounced in older children and in children with a worse growth response to rhGH therapy compared to younger good responders. Those findings are largely in line with our previous observations [14], but the present study was based on the analysis of a larger group of children who were in long-term treatment. 

The effects of rhGH therapy on thyroid function and thyroid hormone metabolism have been previously analyzed by several authors not only in GH-deficient children [5,12,14,15,17,18,20,23,26,27,31,32] and adults [1,6,19,22,24,28,33,34,35,36,37,38] but also in healthy athletes and body builders [39,40]. The number of studies evaluating thyroid function in pediatric GH-deficient patients published in the last twenty years is limited. The results of those analyses are difficult to assess due to the diversity of the studied groups, differences in patients’ age and pubertal development, etiology of GHD and different study protocols. Moreover, the observation period does not usually exceed the first two years of rhGH therapy. Smyczyńska et al. [27], who evaluated TSH and fT4 levels in a mixed group of children treated with rhGH for GDH, GH neurosecretory dysfunction and partial GH inactivity (inactGH), found a significant decrease in fT4 levels in the first year of treatment irrespective of the etiology of the growth hormone disorder without significant changes in TSH levels. The authors reported that a large percentage (22%) of the study group developed hypothyroidism after the initiation of rhGH therapy, which resulted in the implementation of L-thyroxine substitution in those children. They also noticed that the growth response in L-thyroxine-treated children was significantly lower than in patients who remained euthyroid despite a similar increase in IGF-1 secretion during rhGH therapy [27]. Our results confirmed that children with a higher fT4 decrease during rhGH therapy had a worse growth response to rhGH therapy. Those effects were visible not only in the first year of rhGH therapy but also in subsequent years. The study by Keskin et al. [32] based on a homogeneous group of children with idiopathic GHD revealed that rhGH therapy could lead to significant decreases in both fT4 and TSH levels, but hypothyroidism was not diagnosed in any patients in the study group. The authors also analyzed thyroid volume and did not find any changes during rhGH therapy. Unfortunately, a severe limitation of that study is the very small number of studied children and quite a short, one-year observation period [32]. Seminara et al. [21] evaluated thyroid function in GH-deficient children over a longer span of the first two years of rhGH therapy, but the number of patients enrolled in the analysis was scarce. The results of that study showed significant increases in both total T3 (TT3) and fT3 levels after the first year of rhGH therapy, with significant decreases in total T4 (TT4) and fT4 levels in that same period. In the second year of treatment, TT3 and fT3 significantly decreased, which coincided with increases in TT4 and fT4 values. The levels of TSH were unaffected by rhGH therapy. Seminara et al. postulated that the alterations in thyroid function observed after the initiation of rhGH therapy are related to changes in the peripheral metabolism of thyroid hormones, which seem to be transient and disappear in the second year of treatment [21]. These observations are in contrast to our results, which did not confirm the transient character of the fT4 decrease during long-term rhGH therapy. In a more recent study by Yao et al. [15], the observation period lasted from six months to the end of the second year of rhGH therapy, but among the enrolled GH-deficient children, only 16 individuals had a two-year follow-up. The authors reported a significant decrease in fT4 levels after the first year of therapy, with further decreases in the second year of therapy. Simultaneously, they noticed a significant increase in TSH values as early as after six months of rhGH therapy, which then remained higher than baseline until the end of the observation. None of the studied children developed hypothyroidism requiring L-thyroxine therapy [15]. Our study also revealed increases in TSH values after the initiation of rhGH therapy but without any statistical significance. On the other hand, a three-year follow-up study by Ciresi et al. [12], including more than 100 GH-deficient children, showed that after the initiation of rhGH therapy, only fT3 levels significantly increase without any changes in fT4 and TSH values. Moreover, the authors reported that the fT3 increase is more pronounced in patients with a severe GH deficit at baseline [12]. In the present study, based on a similar number of individuals, we did not find any associations between either the baseline maximum GH peak in diagnostic tests or baseline IGF-1 SDS and thyroid function alterations observed during rhGH therapy. 

The results of all those studies do not fully explain the mechanisms of changes in thyroid hormone levels observed in GH-deficient children after the initiation of rhGH therapy. Significant decreases in fT4 values are reported in almost all papers in the field, but the results of the analysis of TSH levels are conflicting. The number of studies evaluating T3 and fT3 values is limited, the number of patients enrolled in the analysis is scarce, and the results are contradictory [12,18,20,21,23,31,37,41]. The data available in the literature indicate that in pediatric and adult populations without GHD, rhGH administration either does not lead to changes in thyroid hormone levels [42] or increases in T4 to T3 conversion in peripheral tissues [10,43]. In patients with both an idiopathic and acquired organic GH deficit, an increase in T4 to T3 conversion also seems to be the main mechanism of the changes in thyroid hormone levels after the introduction of rhGH therapy, but the disclosure of central hypothyroidism, earlier masked by untreated GHD, has also been reported [22,23,24,26,28,44]. In our study, the slight, however significant, depression of fT4 levels in the response to rhGH therapy without significant TSH changes may rather suggest an inhibition of the hypothalamus–pituitary–thyroid axis than alterations of peripheral thyroid hormone metabolism. In the pediatric population, van Iersel et al. [26] showed in a large retrospective cohort study that rhGH therapy could unmask central hypothyroidism, especially in those initially classified as non-acquired apparent isolated GH-deficient children who had congenital structural pituitary abnormalities. Regardless of the cause of thyroid function alterations during rhGH therapy, most authors agree that there is a need for the regular monitoring of thyroid hormone levels in all GH-deficient children after the initiation of rhGH therapy in order to early identify and correct the possible thyroid function abnormalities that could occur during therapy [5,13,14,15,23,29]. Thyroid hormones directly stimulate the transcription of *GH, IGF-1* and insulin-like growth factor binding protein-2 *(IGFBP-2)* and insulin-like growth factor binding protein-4 *(IGFBP-4)* genes, as well as increasing the mRNA of GH and growth hormone-releasing hormone (GHRH) receptors [45,46]. Moreover, Ocaranza et al. [47] reported evidence of direct stimulation of the GH receptor (GHR) by thyroxine in a skin fibroblast culture, in which the simultaneous addition of GH and thyroxine increased signal transduction in the GHR/Janus kinase 2/signal transducer and the activator of the transcription 5 (GHR/JAK2/STAT5) pathway. In line with this data are the observations of Wang et al. [48] in children with idiopathic short stature treated with rhGH, in whom the additional treatment with L-thyroxine improved growth velocity. Such an intervention might be crucial for achieving optimal growth effects in those patients. Our results are in line with those findings. 

In conclusion, we found that the fT4 decrease observed in initially euthyroid children as early as after the first six months of rhGH therapy seemed to be rhGH dose dependent and persisted in the following years of therapy, and children with a higher fT4 decrease achieved a worse growth response during treatment. 

## Figures and Tables

**Table 1:** Characteristics of anthropometric parameters, biochemical parameters, bone age value and rhGH doses.

Parameter	Baseline	6 Months	1 Year	2 Years	3 Years	4 Years
Number of patients	101	101	101	101	101	87
HV (cm/year)	4.9 ± 1.17	-	9.0 ± 1.45	7.7 ± 1.43	6.6 ± 1.66	5.9 ± 1.72
Height SDS	−2.4 (−3.0–(−2.1))	−2.1 (−2.7–(−1.9)) *	−1.9 (−2.4–(−1.6)) *	−1.6 (−2.1–(−1.2)) *	−1.4 (−1.9–(−0.9)) *	−1.2 (−1.7–(−0.8)) *
Weight SDS for HA	−0.3 (−1.8–(−1.0))	−0.4 (−0.6–0.1)	−0.3 (−0.6–0.0)	−0.3 (−0.5–0.1)	−0.3 (−0.5–0.1)	−0.3 (−0.5–0.1)
BMI SDS for HA	−0.6 (−1.0–(−0.1))	−0.5 (−0.8–0.2)	−0.3 (−0.8–0.1)	−0.3 (−0.7–0.1)	−0.3 (−0.6–0.2)	−0.3 (−0.6–0.2)
TSH (µIU/mL)	1.88 (1.38–2.53)	1.88 (1.31–2.27)	2.01 (1.64–2.75)	1.75 (1.28–2.23)	1.57 (1.17–2.04)	1.85 (1.39–2.27)
fT4 (ng/dL)	1.03 (0.97–1.11)	0.97 (0.9–1.05) *	0.98 (0.88–1.05) *	0.92 (0.86–1.02) *	0.92 (0.87–1.0) *	0.92 (0.86–0.97) *
IGF-1 SDS for BA	−0.37 (−0.97–0.15)	1.89 (0.47–3.79) *	1.54 (0.63–2.94) *	1.73 (0.51–2.95) *	1.41 (0.47–2.77) *	1.51 (0.25–2.64) *
BA (years)	7.0 (4.0–10.0)	-	8.5 (5.75–11.5) *	10.0 (6.75–12.5) *	11.5 (8.5–13.5) *	12.5 (9.0–14.0) *
rhGH (µg/kg/week)	-	0.179 (0.174–0.188)	0.179 (0.174–0.188)	0.184 (0.172–0.196)	0.19 (0.181–0.211)	0.2 (0.183–0.218)

Data are presented as mean ± standard deviation (SD) or median with interquartile range as appropriate. * *p* = 0.01 vs. baseline; rhGH—recombinant human growth hormone; HV—height velocity; SDS—standard deviation score; HA—height age; BMI—body mass index; TSH—thyroid-stimulating hormone; fT4—free thyroxine; IGF-1—insulin-like growth factor-1; BA—bone age.

**Table 2:** Characteristics of changes (Δ) in height SDS, IGF-1 SDS and fT4 values after the initiation of rhGH therapy.

Parameter	Δ 1year-Baseline	Δ 2years-Baseline	Δ 3years-Baseline	Δ 4years-Baseline
Height SDS IGF-1 SDS for BA fT4 (ng/dL)	0.6 (0.4–0.8)	1.0 (0.6–1.2)	1.2 (0.9–1.5)	1.4 (1.0–1.7)
	2.06 (0.71–4.03)	2.0 (1.01–3.39)	1.76 (0.44–2.96)	1.44 (0.56–2.39)
	−0.07 (−0.13–0.0)	−0.10 (−0.17–(−0.04))	−0.10 (−0.19–(−0.03))	−0.13 (−0.21–(−0.03))

Data are presented as median with interquartile range. SDS—standard deviation score; IGF-1—insulin-like growth factor-1; BA—bone age; fT4—free thyroxine.

**Table 3:** Associations between changes (Δ) in fT4 levels in the first three and four years of rhGH therapy and changes in IGF-1 SDS values after the initiation of treatment.

Parameter	Δ fT4 3years-Baseline	Δ fT4 4years-Baseline
Δ IGF-1 SDS 1year-baseline	ns	ns
Δ IGF-1 SDS 2years-baseline	R = 0.24, *p* = 0.033	ns
Δ IGF-1 SDS 3years-baseline	ns	R = 0.29, *p* = 0.016
Δ IGF-1 SDS 4years-baseline	ns	ns

fT4—free thyroxine; IGF-1—insulin-like growth factor-1; SDS—standard deviation score; ns—not significant.

## Data Availability

The data underlying this article were collected from medical records and cannot be shared publicly to maintain the privacy of individuals who participated in the study. The data will be shared upon reasonable request to the corresponding author if appropriate.
